# Assessment of Age Effects on Ovarian Hemodynamics Using Doppler Ultrasound and Progesterone Concentrations in Cycling Spanish Purebred Mares

**DOI:** 10.3390/ani11082339

**Published:** 2021-08-08

**Authors:** Francisco Requena, María Joana A. P. M. Campos, Andrés Luis Martínez Marín, Rocío Camacho, Rosa M. Giráldez-Pérez, Estrella I. Agüera

**Affiliations:** 1 Departamento de Biología Celular, Fisiología e Inmunología, Universidad de Córdoba, Ctra. Madrid-Cádiz km 396, 14071 Córdoba, Spain; v02redof@uco.es (F.R.); m92caagr@uco.es (R.C.); rgiraldez@uco.es (R.M.G.-P.); 2 Departamento de Producción Animal, Universidad de Córdoba, Ctra. Madrid-Cádiz km 396, 14071 Córdoba, Spain; z72macaj@uco.es (M.J.A.P.M.C.); pa1martm@uco.es (A.L.M.M.)

**Keywords:** Spanish purebred, power doppler, reproduction

## Abstract

**Simple Summary:**

Power Doppler is a non-invasive imaging technique that allows complete monitoring of the ovary changes in cycling mares. We use Power Doppler to investigate differences in follicular diameter and corpus luteum area as well as in follicular and corpus luteum blood flows between young and mature Spanish Purebred mares. Young mares had higher follicular and corpus luteum blood flows as well as higher blood progesterone levels. Moreover, we found that blood progesterone levels could be predicted in both groups from corpus luteum blood flow with moderate precision and accuracy. These results support the usefulness of Power Doppler to monitor ovarian hemodynamics and the suitability of corpus luteum blood flow to estimate blood progesterone levels in cycling mares.

**Abstract:**

In equine reproduction, accurate and timely detection of the moment of ovulation is of great importance. Power Doppler ultrasound technology is a non-invasive method that enables to assess the morpho-echogenic features and blood flow changes during the estral cycle in mares. The objective of the present study was to evaluate the influence of age on ultrasonographic parameters (follicular diameter, follicular blood flow—FBF, corpus luteum (CL) area and corpus luteum blood flow—CLBF) and blood plasma progesterone concentrations in cycling Spanish Purebred mares (15 less than 8 years old and 15 equal o higher than 8 years old). The ultrasound images obtained were analyzed with the Image Colour Summarizer software, which allows the quantification of the pixels of each image. Young mares had significantly higher FBF, CLBF and plasma progesterone levels. Moreover, linear regression analysis showed that blood progesterone levels could be predicted in both groups from CLBF with moderate precision and accuracy. In conclusion, Power Doppler was useful to assess ovarian hemodynamics. Our results support that age is a factor that significantly influences FBF and CLBF as well as blood progesterone concentration in mares. More studies would be needed to develop high precision and accuracy predictive models of blood progesterone concentration from CLBF measured by Power Doppler.

## 1. Introduction

In equine reproduction, accurate and timely detection of the moment of ovulation is of great importance for the following reasons: (i) to ensure that ovulation occurred within the estimated time period after pre-ovulation; (ii) to establish the number of ovulations in relation to the number of pre-existing follicles, to be able to carry out the appropriate management in cases of twins, and (iii) to ensure the rupture and collapse of the follicle, followed by the release of the oocyte, thus allowing to detect possible cases of anovulatory follicles [1]. There is an increased permeability of blood vessels and an intense angiogenesis in the ovary during follicular development, ovulation, and subsequent corpus luteum (CL) formation.

Power Doppler ultrasound has the advantage of being a non-invasive method that enables to assess not only the morpho-echogenic features but also physiological events during the estral cycle such as the vascularization and blood flow (arterial and venous) of ovaries [2]. Measurement of follicle and CL blood flow (CLBF) can be achieved by this technique during the right-hander and also after the administration of hormonal treatments [3], which allows a deeper understanding of the reproductive pathophysiology of a mare. To make an in-depth study of the ovary, a useful tool to evaluate CL activity is to determine the blood concentration of progesterone [4]. Accordingly, any relationship between blood progesterone concentration and CLBF could be a predictive model to estimate the functionality of CL [5] avoiding stressful situations for animals [6] and providing a good prognosis and improving mare care and their practice management [7]. Power Doppler could contribute to the enhancement of equine reproductive management, achieving better use of genetic material and increasing financial return [8].

The Spanish Purebred is the most representative autochthonous equine breed in Spain. It has experienced a great boom in recent years and is widespread worldwide, so it is essential to guarantee its preservation by the Spanish State, as guarantor of a genetic heritage that cannot suffer any deterioration, avoiding possible risks of the dispersion of criteria that could compromise its adequate conservation [9]. A qualified breeding mare is three or more years old and meets the basic aptitude for reproduction, according to the established criteria referring to the breed prototype or morphology, functional test and examination of the reproductive system and veterinary control [9].

The objective of the present study was to evaluate the influence of age on ultrasonographic parameters (follicular diameter, follicular blood flow (FBF), CL area and CLBF) and blood plasma progesterone concentrations.

## 2. Materials and Methods

### 2.1. Animals

Cycling Spanish Purebred mares (n = 30; 600–700 kg), with a range of age between 4 and 19 years (average 9.3 years) were evaluated. The animals maintained a high body condition score (score ≥ 8) during the experiment [10]. All mares had normal reproductive tract, typical oestrus cycles length and were free from infectious diseases. All mares were housed in stables with ab libitum access to water, hay and mineral salt. Mares were divided into two groups: young (<8 years, n = 15) and old (≥8 years, n = 15). The data collection was carried out in the “Miguel Ángel de Cárdenas” stud farm and located at San Pablo Farm (Écija, Seville, Spain). Animal care was fully in compliance with the University of Cordoba requirements on animal welfare and experimentation (2021PI/19).

### 2.2. Mare Management

All mares in this study underwent ovulation induction by administering a single IV injection of one dose of 3000 IU hCG (VETERIN CORION^®^). In all of them, the appearance of ovulation was recorded between 24- and 48-h post-administration by ultrasound. When the follicular diameter was ≥35 mm, endometrial edema was verified by ultrasound and consistent cervical tone by rectal palpation [11].

Artificial insemination was carried out with refrigerated semen obtained from stallions from the herd itself. Pregnancy diagnosis was not included. In this study, it was decided to avoid sedation of the females to prevent the possible vasodilator effects of the sedative, although it minimizes artifacts in the color Doppler video recording.

### 2.3. Ultrasonography

**Figure 1:**
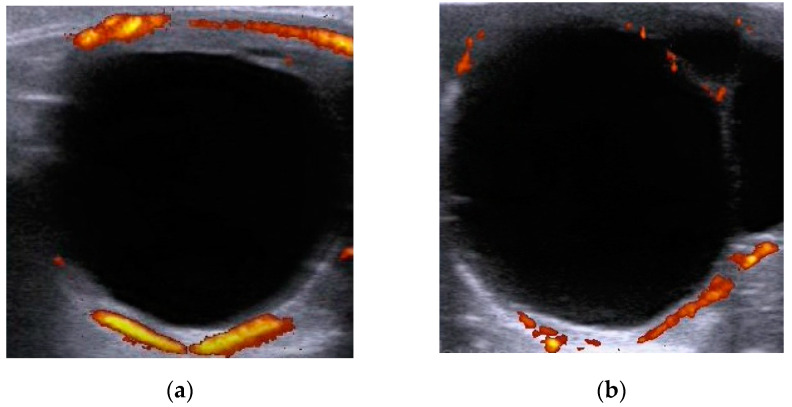
Representative image of preovulatory follicles, obtained at the time of ovulation induction, using Power Doppler ultrasound. (**a**) Blood flow follicle of a young mare; (**b**) Blood flow follicle of an aged mare.

The ultrasound examinations (US) were performed by the first author, with a US-B-mode and Doppler using the MyLabOneVet ultrasound (ESAOTE, Barcelona, Spain). The device was equipped with a 6–10 MHZ multifrequency linear transducer. All the US were performed by the same person to minimize the possible differences in the uptake and, consequently, the interpretation of the ultrasound images. The images were always captured with the same settings (10 MHz frequency, 70% mode B gain and 40% Power Doppler gain) and were recorded for later analysis on the same ultrasound machine. For Power Doppler imaging, a 30-s video was made of each structure from which 3 representative images were selected. Using the online version of Image Colour Summarizer^®^ software, the chosen images were analyzed. This software allows the quantification of the percentage of colored pixels existing in an image, representative of ovarian blood flow. This technique transforms a qualitative analysis into a quantitative one. The user only has to introduce the file and process the selected image. Through the sum of all the colored pixels, the percentage of existing blood flow is obtained. The recorded Power Doppler images were taking in mares with preovulatory follicles ≥35 mm (Figure 1), which coincided with the administration of hCG (ovulation induction). The ovulation time was assumed between 36–40 h post-administration of hCG. The CL examination was 5.83 ± 0.62 and 6.40 ± 0.54 days in young and old age groups, respectively. 

Care was taken to do detailed examinations of the ovaries and uterus allowing a correct interpretation of the follicular dynamics. Along with the examination of both ovaries, images of the uterus were also recorded for a more complete analysis of the mare’s reproductive dynamics.

In order to minimize possible errors and make the image of each ultrasound moment as representative as possible, the average of the parameters evaluated (diameter of the preovulatory follicle, percentage of FBF, area of the CL and percentage of CLBF) in the 6 images recorded from the total number of mares was evaluated. The mean value of the four ultrasound parameters studied was used for the statistical analysis.

### 2.4. Progesterone Assay

Jugular vein blood samples were obtained using heparinized 4 mL vacutainers, centrifugated (1000× *g* for 10 min at 18–25 °C) and plasma decanted and stored (−20 °C) until progesterone assay. Progesterone was evaluated with the commercial ELISA method EIA-5223 (DRG Instruments Gmbh, Marburg, Germany) which is specifically dedicated to equine [12]. 

### 2.5. Statistical Analysis

SAS University Edition 3.8 (SAS Institute, Cary, NC, USA) was used in the statistical analysis. The GLM procedure was used to investigate differences in ultrasound parameters and blood plasma progesterone concentrations due to age. Regression analysis in the GLMSELECT procedure was used to investigate the relationship between progesterone concentrations and CLBF. Model performance was assessed by the coefficient of determination (R2), the root of the mean square of prediction error (RMSPE), the RMSPE expressed as a proportion of the observed mean (%RMSPE), and the concordance correlation coefficient (CCC). Furthermore, the mean square of prediction error (MSPE) was decomposed into mean bias (measure of precision), slope bias (measure of accuracy), and random error [9]. Statistical significance was declared at *p* < 0.05.

## 3. Results

All of the 30 mares ovulated within the first 48 h (38.8 ± 1.3 h) after hCG treatment. The results from follicular and CL measurements and blood plasma progesterone concentrations are presented in Table 1. Blood plasma progesterone concentrations denoted that CL was functional.

Table 2 shows the comparison between both age groups. Young mares had significantly higher FBF (*p* < 0.05), CLBF (*p* < 0.001) and plasma progesterone levels (*p* < 0.001). The CL area and follicular diameter were non significant (*p* > 0.05).

The best regression models obtained in the present study for predicting progesterone concentrations from CLBF data shown in Table 1 are presented in Equation (1) (all data), (2) (young mares) and (3) (aged mares).

P_4_ (ng/mL) = −0.392 (±1.1418) + 0.369 (±0.0593) × CLBF R^2^ = 0.58; RMSPE = 1.01 ng/mL; %RMSPE = 16.2 %; CCC = 0.73 *p* < 0.001 (1)

P_4_ (ng/mL) = 4.878 (±0.9960) + 0.1555 (±0.0470) × CLBF R^2^ = 0.46; RMSPE = 0.33 ng/mL; %RMSPE = 4.0 %; CCC = 0.63 *p* < 0.01 (2)

P_4_ (ng/mL) = 2.966 (±0.7313) + 124 (±0.0428) × CLBF R^2^ = 0.39; RMSPE = 0.49 ng/mL; %RMSPE = 9.7 %; CCC = 0.56 *p* < 0.05 (3)

Three models were significant and showed moderate precision, moderate to high accuracy, and moderate to weak reproducibility according to R^2^, RMSPE, %RMSPE and CCC values. Furthermore, precision and accuracy were reinforced by the fact that none of the models showed mean or linear bias, with nearly 100% of MSE due to random error. The best equation in terms of precision and reproducibility was Equation (1), but it had less accuracy than the other two equations.

## 4. Discussion

It is common to find aged mares in herds for their genetic value and for their performance. Reproductive aging in mares is a physiological process that affects their fertility; thus, it must be understood how reproductive parameters change with age [13]. The association between Power Doppler and hormonal measurements throughout the mare’s oestrus cycle could allow to evaluate the reproductive ability in mares [14,15]. Power Doppler is a non-invasive technique that permits the visualization of the internal organs. It has the advantage of being non-disruptive to functions, without the risk of exposure to X-ray radiation, and it allows frequent use, not only in isolated reproductive organs, but also the monitoring of complete reproductive events and ovarian hemodynamics [11,16,17].

In the current study, Power Doppler allowed us to find significant differences in the follicular and CL vascular perfusion between young and aged mares 5–7 days after ovulation in concordance with Campos [8]. Our results agree with Bollwein et al. [4], Ginther et al. [18] and Alonso et al. [19], who reported maximum vascularization between 6-days and 8-days post-ovulation. It is well known that luteal vascularization is important in the supply of the nutrients and substrates required for the optimal function and maturity of the CL [20]. 

No differences were detected regarding follicle diameter and CL area in agreement with previous studies [21,22]. Consistent with those authors, age is a critical factor that influences the quantity and quality of the equine preantral follicle. 

The concentrations of progesterone obtained denoted that corpora lutea were functional in all mares. The blood plasma progesterone levels quantified in this study support that the young mares CL, from the fifth day after ovulation, had the functional ability to preserve the pregnancy until the placenta assumes its function [17]. Although aged mares had lower progesterone concentrations than younger ones, the values were higher than the minimal plasmatic level (4 ng/mL). Therefore, a low reproductive performance would not be expected in the aged mares of the current study [23]. Again, the physiological cause of the significant differences in plasma progesterone concentration between aged and young mares would be related to changes in characteristics of reproductive cycles [24]. Aging in mares affects the development of the dominant follicle. Moreover, higher postovulatory plasma progesterone concentrations in young vs. aged mares have been previously reported [25,26]. Although we used hCG treatment for ovulation induction, we did not establish the correlation between that treatment and progesterone concentrations. A study performed by Alonso et al. [19] suggested that hCG treatment did not rise progesterone concentrations. 

In the present study, CLBF was found to be a moderately good predictor of plasma progesterone levels. These results would support the interest in Power Doppler as a non-invasive technique to quantify plasma progesterone levels in cycling mares. Moreover, CLBF and progesterone levels in mares have been found to be positively correlated [4,27]. Using the data presented by Ishak et al. [27] in Equation 1, plasma progesterone levels would be underpredicted by 21% from CLBF, which suggests that this kind of prediction needs refinement. 

Sales et al. [17] found that Doppler ultrasound correlates with the progesterone concentration and the embryo transfer day in Mangalarga Marchador mares, which indicates that Doppler ultrasound could be an important tool in the selection of appropriate embryo recipient mares in Spanish Purebred. 

## 5. Conclusions

Our results support that age is a factor that significantly influences FBF and CLBF as well as the plasma progesterone concentration in mares. Power Doppler was useful to assess ovarian hemodynamics. Progesterone plasma levels could be predicted from CLBF measured by power Doppler. More studies are needed to enhance the precision and accuracy of such predictions.

## Figures and Tables

**Table 1:** Mean ± standard deviation (SD), minimum (Min) and maximum (Max) values of the follicle and corpus luteum ultrasonic parameters and blood plasma progesterone concentration from mares included in the study (n = 30).

	Age Group								
Parameters	Old			Young			Total		
	Mean ± SD	Min	Max	Mean ± SD	Min	Max	Mean ± SD	Min	Max
Follicular diameter (mm)	42.12 ± 2.58	38.40	46.30	43.67 ± 2.94	38.60	49.00	42.89 ± 2.83	38.40	49.00
Follicular blood flow (%)	6.63 ± 1.31	4.20	8.53	8.00 ± 1.59	5.68	11.20	7.31 ± 1.59	4.20	11.20
Corpus luteum area (mm)	7.12 ± 1.27	4.38	8.63	7.79 ± 1.23	4.38	9.50	7.46 ± 1.27	4.38	9.50
Corpus luteum blood flow (%)	16.78 ± 3.29	12.21	22.53	21.09 ± 20	16.65	23.85	18.94 ± 3.46	12.21	23.85
Progesterone (ng/mL)	5.04 ± 0.65	4.20	6.30	8.16 ± 0.46	7.36	8.90	6.60 ± 1.68	4.20	8.90

**Table 2:** Least squares mean of ultrasonic parameters and progesterone concentrations.

	Age Group			
Parameters	Old	Young	SEM ^1^	*p*
Follicular diameter (mm)	42.12	43.67	0.51	0.13
Follicular blood flow (%)	6.63	8.00	0.29	<0.05
Corpus luteum area (mm)	7.12	7.79	0.23	0.15
Corpus luteum blood flow (%)	16.78	21.09	0.63	<0.001
Progesterone (ng/mL)	5.04	8.15	0.30	<0.001

^1^ Standard error of the mean.

## Data Availability

The data presented in this study are available in the article.
